# Evaluation of Postpartum Midwifery Care in Greece Based on Patient-Reported Outcomes Using the MMAYpostpartum Questionnaire: A Cross-Sectional Study

**DOI:** 10.7759/cureus.46129

**Published:** 2023-09-28

**Authors:** Alexandra Kosiva, Emmanouil M Xydias, Apostolos C Ziogas, Elias Tsakos, Ioannis Thanasas, Aikaterini Lykeridou

**Affiliations:** 1 Department of Midwifery, School of Health and Care Sciences, University of West Attica, Athens, GRC; 2 Department of Obstetrics and Gynaecology, EmbryoClinic IVF, Thessaloniki, GRC; 3 Department of Medicine, University of Thessaly, School of Health Sciences, Larissa, GRC; 4 Department of Obstetrics and Gynecology, General Hospital of Trikala, Trikala, GRC

**Keywords:** patient-reported outcomes, questionnaire study, midwifery care, postpartum care, observational cross-sectional study

## Abstract

Background

The midwife’s role throughout pregnancy and delivery management is essential, with multiple healthcare systems even following a midwife-led model of care. Of particular interest is the improvement and optimisation of midwifery postpartum care, which in Greece is empirically known to have decreased in quality, both due to the economic crisis and the recent pandemic.

Aims

To collect patient-reported outcomes with regard to the quality of midwifery services in Greece, ascertain baseline patient characteristics that may affect quality assessment, identify key areas for improvement, and propose patient subgroups who would most benefit from more specialized care.

Setting and design

A prospective, cross-sectional, questionnaire-based survey using the Measurement of Midwifery quality postpartum (MMAYpostpartum) questionnaire was conducted in public and private postpartum care centers in Greece.

Methods

The MMAYpostpartum questionnaire was distributed to 316 eligible women who received postpartum midwifery care in a healthcare center in Greece during the past three years. Multivariate linear regression was performed to examine significant correlations between baseline parameters and questionnaire scores.

Results

Ultimately, 204 answers were collected and analyzed. The patient's mean age was 35.5 years, and the mean body mass index (BMI) was 23.5. Overall, submitted scores were lower than those observed in the literature. A statistically significant correlation between older age, delivery at a public hospital, a history of hospitalization during pregnancy, and a lower midwifery service score was demonstrated. No other factors had a statistically significant effect on the quality score.

Conclusions

Delivery at public healthcare centers, older maternal age, and a history of hospitalization during pregnancy are significant predictors of a lower perceived quality of midwifery care. Thus, such patient subgroups may constitute potential targets for more meticulous midwifery care when resource setting prohibits the overall improvement of quality. Further research is required to collect additional data on patient insight and to test the present observations in a clinical setting.

## Introduction

The midwife is defined as an essential and qualified professional who provides support, care, and advice to women during pregnancy, labor, and the postpartum period, as well as general female and reproductive healthcare [1,2]. Historically, they have been trained and/or highly experienced practitioners, indispensable in the care of prospective mothers from antiquity and ancient Greece [3] to the Middle Ages [4] and eventually through the Modern Era. With a few historical exceptions, the importance of establishing a strong and influential relationship between patient and midwife has been repeatedly highlighted in both historical and modern literature [4].

Midwife contribution is particularly significant during the early postpartum period since it is a particularly anxiety-inducing period for the mother [5], with additional psychological and physiological effects being noted by multiple studies [6-8]. Effects on breastfeeding behavior, thus indirectly on the growing infant as well, have also been noted in caesarean section deliveries in particular [9]. However, early postpartum support, with the establishment of a healthy patient and care-provider relationship and the provision of useful, practical information, has been shown to mitigate these effects and greatly assist the patient in her new role [9]. These observations of the significance of postpartum care have led to many advocating for midwife-led maternity care as well as midwife continuity care models, a prospect shown to offer multiple advantages and considerable patient satisfaction, further highlighting the significance of the midwife’s contribution [10,11].

However, such models of care require adequate resources and personnel, prerequisites not always fulfilled in all settings. With regard to public Greek healthcare, following the economic recession of 2008, public hospital budgets have been reduced, supply shortages have been repeatedly reported, and a considerable increase in the prevalence of emotional exhaustion, depersonalization, and a low sense of personal accomplishment amongst healthcare workers was observed [12]. This adverse situation was further exacerbated by the sudden, worldwide spread of the COVID-19 pandemic, which severely increased the healthcare workload of the already understaffed hospitals, with healthcare workers needing to work to exhaustion to compensate. This stressful environment invariably led to an increased incidence of physical and mental fatigue, burnout, and depression, as noted by multiple studies [13,14].

The aim of this study is to assess the quality of midwifery care in Greece during these challenging times and identify key weaknesses in the current model based on patient-reported outcomes. An additional aim is to identify patient factors that may predict which patients are in need of more particular care and thus assist in the improvement of overall performance at a lower resource cost compared to large-scale changes.

## Materials and methods

Study design

In order to examine the above research questions, a cross-sectional study design was employed. The validated Measurement of Midwifery quality postpartum (MMAYpostpartum) questionnaire by Peters et al. (2021) [15] was utilized to record and quantify participant perspectives. Printed handouts or digital participation links were distributed individually to eligible participants. Data was collected and analyzed. All patients provided informed written consent for participation in the study. This manuscript has been prepared in accordance with the strengthening of the reporting of observational studies in epidemiology (STROBE) statement for cross-sectional studies [16].

Setting

The questionnaire was distributed in printed and digital form from September 2022 to December 2022 in public and private postpartum care centers in Greece. Digital questionnaires were drafted using the Google Forms online survey platform. The data was collected anonymously. The study was designed and approved and the data were analyzed at the Department of Midwifery, University of West Attica, Athens, Greece.

Participants

Eligible for participation were women who delivered within the past three years in a hospital or other specialized medical center in Greece. Women delivering at home were excluded from this survey. Women with pregnancies resulting in stillbirths were excluded from this survey. Only the midwifery services provided during the women's stay at the hospital were assessed. Additional services at home by the same or other midwifery personnel were beyond the scope of this survey. All participants provided informed consent for participation in the survey and publication of the results. All eligible participants accessible by the investigative team were invited to participate.

Questionnaire

For the purposes of this cross-sectional survey, a questionnaire was prepared in order to assess patients’ perspectives on the quality of midwifery postpartum care. To facilitate this assessment, the validated MMAYpostpartum questionnaire by Peters et al. (2021) [15] was utilized. The MMAYpostpartum questionnaire assesses patient perspectives on midwifery care using 16 questions organized in three categories, or scales, based on the main target parameter of care for each question. The first scale, "personal control,", contains three questions and focuses on patient involvement in decision-making and whether their personal sovereignty and independence were respected. The second scale, "trusting relationship", contains seven questions and measures the midwife’s empathy, understanding, and respect for the patient’s individual physical and emotional needs, vital prerequisites for developing a trusting midwife-patient relationship. The third and final scale, "orientation and security", contains six questions and measures the practical advice and medical information that the midwife provides in order to assist in the patient’s new role and responsibilities. All 16 MMAYpostpartum questions were scored on a five-point Likert scale, using a neutral center option. Namely, the possible answers were "not applicable at all," "not applicable," "neither," "applicable," and "fully applicable" [17].

In addition to the MMAYpostpartum items, additional questions were added to assess participant baseline characteristics and to later be used to test for possible correlations. Such characteristics included patient age, BMI, type of healthcare (state or private), education level, parity, type of pregnancy (single or multiple), occurrence of pregnancy disorders, hospitalization during pregnancy, delivery timing (pre-term/full-term), delivery mode, post-partum hospitalization duration, breastfeeding, and the main midwifery care parameter in need of improvement. Education level was assessed based on the International Standard Classification of Education (ISCED) 2011 [18]. Pregnancy disorders were specified as gestational diabetes, pre-eclampsia, in utero growth restriction (IUGR), etc. Overall, the questionnaire included 30 questions, along with a consent question for participation and anonymous publication of the results, which was a mandatory prerequisite for participation.

The primary outcome assessed was the MMAYpostpartum score and its correlation to baseline patient characteristics, so that predictive factors for the final score could be established. Secondary outcomes were correlations of baseline characteristics to the scores of the three MMAYpostpartum subscales individually.

Bias minimization

In order to minimize overall survey bias, several steps were taken. Firstly, an already tested and validated questionnaire was employed for data acquisition and quantification, and any additional questions were structured in a clear, concise way and according to the multiple-choice format in order to minimize overall response bias. Secondly, the questionnaire was administered in written form (printed or digital) and was not completed under the immediate supervision of the investigators. This measure ensured the minimization of interviewer bias while maintaining the availability of the investigator to provide instructions and clarifications when necessary. Thirdly, no identifying information was recorded on the questionnaires; digital forms were completed and submitted to the participant’s private electronic device; and printed forms were handed out folded. This ensured that the investigator would not be privy to submitted answers, thus minimizing social desirability bias.

Data collection and analysis

Patient answers were collected, and data extraction was performed by two investigators. Written data was converted to digital form. Missing values were not inferred, and incomplete answers were excluded from the analysis.

Statistical analysis

Linear regression was performed, with the MMAYpostpartum overall score being the dependent variable and the baseline participant characteristics being the independent variables. The collinearity of variables and the standardized beta coefficient for every variable were calculated in order to identify any confounding factors and to ensure that the effect of each variable was assessed individually. The standardized residual statistic was calculated in order to test for outlier values. The R square coefficient of determination was also calculated to quantify the dependent variable variance attributable to the independent variables. Pearson correlations were calculated for the most clinically relevant parameters and assessed for statistical significance. The same methodology was also applied, using the three MMAYpostpartum questionnaire subscale scores as the dependent variable. All analyses were conducted using IBM SPSS Statistics for Windows, Version 26.0 (released 2019; IBM Corp., Armonk, New York, United States).

## Results

From the 316 eligible mothers contacted, 225 completed the questionnaire (71.2% response rate). From the completed questionnaires, 21 were excluded due to missing data. Overall, 204 participants provided complete data and were ultimately included in this survey. These participants were on average 35.5 years of age, with ages covering a wide spectrum, varying from 19 to 53 years. Participants had a mean BMI of 23.5, with individuals ranging from underweight (BMI = 15.8) to morbidly obese (BMI = 49.5). All participants had received at least primary education, with the majority possessing university degrees. Participants were almost evenly matched with regard to parity and healthcare provider type. The majority (55.9%) underwent a caesarean section (CS), with the majority (54.9%) also being hospitalized for three days. When asked to provide key areas for improvement in post-partum midwifery care, the majority of participants indicated that no improvement was necessary, while the second most popular area was psychological knowledge and support, with scientific knowledge being a distant third. Participants were seemingly satisfied with the level of practical expertise of the midwifery staff, while the few individuals who provided a key area of their own mostly stressed the inadequate number of midwifery staff in most facilities. This information is summarized in Table 1. Collected questionnaire scores per question, along with pooled scores per scale and an overall MMAYpostpartum score, are presented in Table 2.

**Table 1:** Summary of the baseline characteristics of the participants SD: standard deviation; ISCED: International Standard Classification of Education

Baseline characteristics		Participants (n=204)
Age mean±SD, median (min-max), years		35.47±6.8, 35.5 (19-53)
BMI mean±SD, median (min-max)		23.5±4.6, 22.3 (15.8-49.5)
Education	Primary- Secondary (ISCED 1-4) n, (%)	52 (25.5%)
	Tertiary- Bachelors degree (ISCED 5-6) n, (%)	104 (50.1%)
	Tertiary- Masters/doctoral degree (ISCED 7-8) n, (%)	48 (24.4%)
Healthcare	State/Public n, (%)	84 (41.2%)
	Private n, (%)	120 (58.8%)
Parity	Primipara n, (%)	97 (47.6%)
	Multipara n, (%)	107 (52.4%)
Pregnancy	Single n, (%)	197 (96.6%)
	Multiple n, (%)	7 (3.4%)
High risk pregnancy	Yes n, (%)	25 (12.3%)
	No n, (%)	179 (87.7%)
Hospitalization during gestation	Yes n, (%)	16 (7.8%)
	No n, (%)	188 (92.2%)
Labour timing	Normal (>37 weeks) n, (%)	188 (92.2%)
	Premature (<37 weeks) n, (%)	16 (7.8%)
Mode of delivery	Spontaneous vaginal n, (%)	85 (41.6%)
	Forceps/vacuum n, (%)	5 (2.5%)
	Caesarean section n, (%)	114 (55.9%)
Post-partum hospitalization duration	Two days n, (%)	14 (6.9%)
	Three days n, (%)	112 (54.9%)
	Four to seven days n, (%)	78 (38.2%)
Breastfeeding	Yes n, (%)	124 (60.8%)
	No n, (%)	80 (39.2%)
Focus-areas for midwifery care improvement	No improvement necessary n, (%)	87 (42.6%)
	Scientific-medical knowledge n, (%)	33 (16.2%)
	Practical skills n, (%)	5 (2.5%)
	Psychological knowledge n, (%)	74 (36.2%)
	Other n, (%)	5 (2.5%)

**Table 2:** Summary of the submitted questionnaire scores per scale and item

Scale	Item	Mean	SD	Median	IQR	Min-max
Scale 1: personal control	Q1) I couldn’t speak freely about my feelings/fears	3.83	1.16	4	2	1 - 5
	Q2) Examinations were performed without consent	4.03	1.10	4	2	1 - 5
	Q3) I felt judged	4.1	1.07	4	1	1 - 5
	Scale 1 overall	11.97	2.80	12	4	3 - 15
Scale 2: trusting relationship	Q4) The midwife was friendly to significant others	4.06	1.00	4	1	1 - 5
	Q5) Information was neutral/judgement free	3.87	0.98	4	2	1 – 5
	Q6) Lifestyle choices were respected	3.95	1.04	4	2	1 – 5
	Q7) Privacy was respected	3.98	1.06	4	1	1 – 5
	Q8) The midwife was organised	3.99	1.10	4	1	1 – 5
	Q9) I received the right information at the right time	3.72	1.21	4	2	1 – 5
	Q10)The midwife took time to listen	3.76	1.22	4	2	1 – 5
	Scale 2 overall	27.33	6.57	28	10	7 - 35
Scale 3: orientation and security	Q11) The midwife helped with physical complaints	4	1.00	4	1	1 – 5
	Q12) The midwife helped me deal with strong emotions	3.63	1.15	4	2	1 – 5
	Q13) The midwife helped me understand what was happening to my body	3.61	1.18	4	2	1 – 5
	Q14) The midwife helped my partner adjust to his/her new role	3.26	1.31	3	2	1 – 5
	Q15) The midwife enabled me to connect with other women and families	2.91	1.30	3	2	1 – 5
	Q16) The midwife respected my religion/ culture	3.94	0.96	4	2	1 - 5
	Scale 3 overall	21.35	5.88	21	7	6 - 30
Overall score		60.66	13.94	63	20	20 - 80

Compared to the observations of Peters et al. (2021) [15], who also used the MMAYpostpartum questionnaire, our data showed significant differences. More specifically, the majority of questions received a lower mean score in the present study compared to Peters et al. (2021) [15], namely Q2 (4.03±1.103 versus 4.41±1.2, p<0.001), Q3 (4.1±1.066 versus 4.42±1.25, p<0.001), Q4 (4.06±0.998 versus 4.73±0.56, p<0.001), Q5 (3.87±0.984 versus 4.25±0.95, p<0.001), Q6 (3.95±1.035 versus 4.59±0.68, p<0.001), Q7 (3.98±1.055 versus 4.62±0.65, p<0.001), Q8 (3.99±1.098 versus 4.29±0.89, p<0.001), Q9 (3.72±1.206 versus 4.27±0.89, p<0.001), Q10 (3.76±1.218 versus 4.52±0.77, p<0.001), Q11 (4±0.995 versus 4.39±0.81, p<0.001), Q12 (3.63±1.148 versus 3.87±1.02, p=0.004), Q13 (3.61±1.179 versus 4.25±0.89, p<0.001), Q14 (3.26±1.305 versus 3.67±1.13, p<0.001). Conversely, Q15 scored higher in this study, compared to Peters et al. (2021) (2.91±1.303 versus 2.52±1.23, p<0.001) [15], while no statistically significant differences between the two studies were observed for Q1 (3.83±1.162 versus 3.89±1.37, p=0.5695) and Q16 (3.94±0.958 versus 3.88±0.98, p=0.4392).

**Figure 1:**
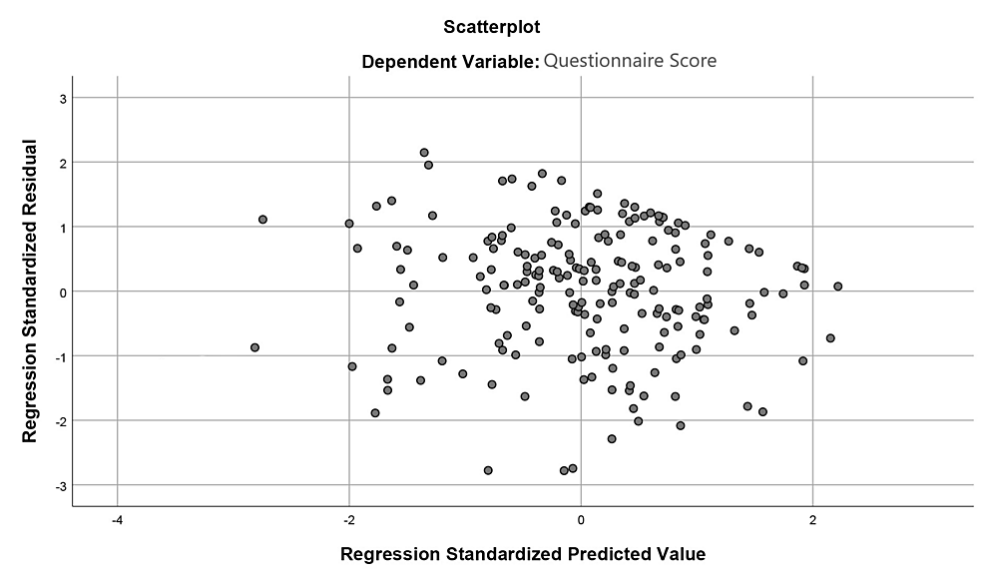
Scatterplot of residuals and predicted values, depicting a random spread pattern

With regard to the correlations observed in the present study alone, linear regression was performed in order to assess any effect of patient baseline characteristics on the overall MMAYpostpartum score. The collinearity of variables was tested and demonstrated not to affect this analysis, with tolerance and variance inflation factor (VIF) values being within acceptable parameters. No outlier values were observed during this analysis, with the standardized residual statistic numbering within acceptable margins (-2.85-2.199), as also demonstrated in Figure 1. The R square coefficient of determination was 0.309, indicating an acceptable percentage of variance in the dependent variable (MMAYpostpartum score) explained by the independent variables (the various assessed patient parameters).

Pearson correlations were calculated for the most clinically relevant parameters and assessed for statistical significance. From the assessed variables, only participant age, healthcare type, and hospitalization during gestation showed a statistically significant correlation, albeit evaluated as low (R=0.3-0.5), based on the available literature [19], while post-partum hospitalization duration also showed a statistically significant correlation, which was however minimal (R<0.3). Furthermore, the standardized beta coefficient was calculated for each parameter in order to assess their individual impact on the overall MMAYpostpartum score. Participant age, healthcare type, and hospitalization still had a statistically significant impact, with BMI also having a marginally significant effect; however, post-partum hospitalization did not retain its statistical significance. All analysis results are summarised in Table 3.

**Table 3:** Summary of the key results of the performed linear regression analysis BMI: body mass index; VIF: variance inflation factor

	Pearson’s R	P - value	Stand. Beta coefficient	P - value	Tolerance	VIF
Age	- 0.346	<0.001	-0.315	<0.001	0.909	1.100
BMI	0.069	0.163	0.129	0.044	0.897	1.115
Healthcare	0.325	<0.001	0.277	<0.001	0.913	1.095
Parity	0035	0.312	0.0001	0.998	0.851	1.176
High risk pregnancy	-0.070	0.160	-0.012	0.854	0.812	1.232
Hospitalization during gestation	-0.309	<0.001	-0.273	<0.001	0.845	1.183
Labour timing	-0.041	0.278	-0.062	0.330	0.900	1.111
Mode of delivery	0.008	0.455	-0.108	0.125	0.739	1.353
Post-partum hospitalization duration	0.131	0.031	0.105	0.121	0.798	1.254
Breastfeeding	-0.023	0.374	-0.013	0.835	0.926	1.080

**Figure 2:**
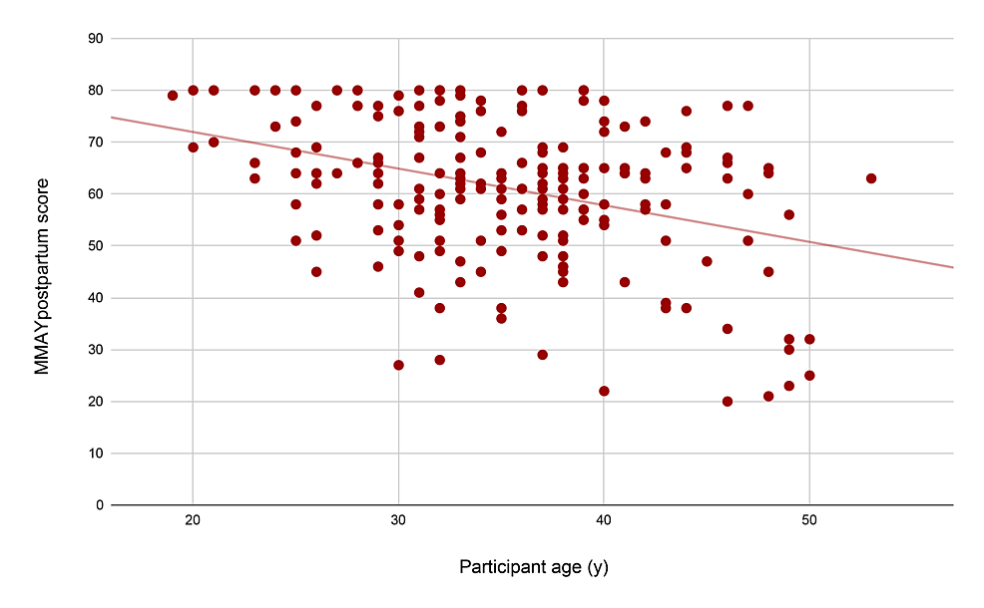
Scatterplot of the MMAYpostpartum score and participant age The trend line indicates a downward trend of score as age increases, which is statistically significant. MMAYpostpartum: Measurement of Midwifery quality postpartum

**Figure 3:**
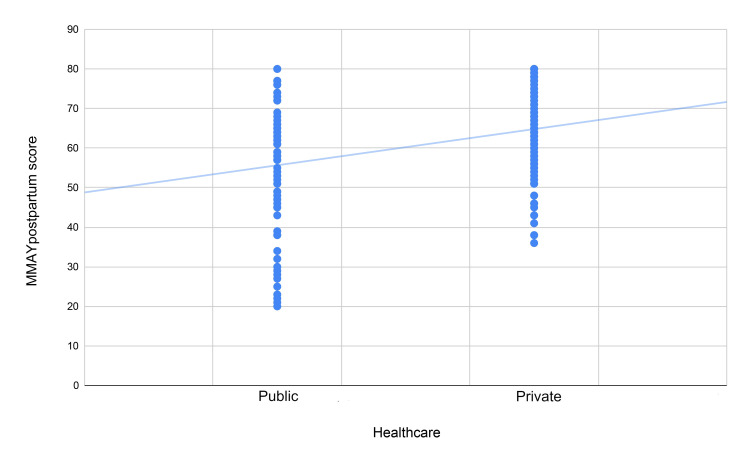
Scatterplot of the MMAYpostpartum score and type of healthcare The trend line indicates an upward  trend of score for women who had received private healthcare, which is statistically significant. MMAYpostpartum: Measurement of Midwifery quality postpartum

**Figure 4:**
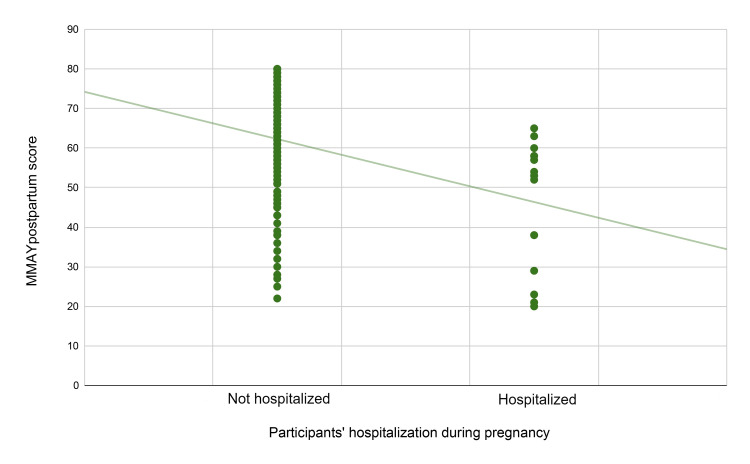
Scatterplot of the MMAYpostpartum score and participant hospitalization during pregnancy The trend line indicates a downward trend for patients who had been hospitalized during their pregnancy, which is statistically significant. MMAYpostpartum: Measurement of Midwifery quality postpartum

With regard to the individual effect of the statistically significant variables, participant age showed a significant negative correlation with MMAYpostpartum score, indicating that older women were not as satisfied with the services provided compared to younger women. In particular, the observed beta coefficient of -0.315 indicates that with every increase in participant age by one standard deviation, the MMAYpostpartum score drops by 0.315 standard deviations. This correlation is visually depicted in Figure 2. Healthcare type also had a significant effect, with private healthcare demonstrating a significant positive correlation to MMAYpostpartum score, indicating that women treated in private centers were more satisfied with the midwifery services provided compared to those in public healthcare. In particular, the observed beta coefficient of 0.277 indicates that for women treated in private centers, the MMAYpostpartum score is increased by 0.277 standard deviations compared to those treated at public centers. This correlation is visually depicted in Figure 3. Hospitalization during pregnancy was a statistically significant factor as well, demonstrating a negative correlation to MMAYpostpartum score, indicating that women who were hospitalized during pregnancy were not as satisfied with the provided postpartum midwifery care. In particular, the observed beta coefficient of -0.273 indicated that for women who were hospitalized during their pregnancy, the MMAYpostpartum score was decreased by 0.273 standard deviations compared to those who were not hospitalized. This correlation is visually depicted in Figure 4.

Linear regression was additionally attempted with the three MMAYpostpartum scale scores in order to assess whether any baseline variables affected any aspect of midwifery services ("personal control", "trusting relationships,", "orientation, and security") more or less than the others. The results of this analysis, however, either showed no deviation from those observed during the overall MMAYpostpartum score assessment or correlations did not maintain their statistical significance.

## Discussion

In this study, we attempted to evaluate the adequacy of midwifery care services in Greece during a long, nationwide period of healthcare resource scarcity and manpower insufficiency. With data representing mothers of a wide range of baseline demographic, physical, clinical, and psychosocial characteristics, we aimed to provide an overall depiction of the current state of midwifery care, areas in need of improvement, and patient subgroups in need of particular attention. Our data showed that the level of midwifery services was evaluated mostly as inferior to that observed in the original study, with a few exceptions. With regard to observations made solely in the present study, when asked directly, most patients evaluated the provided care as adequate and in no need of further improvement, with the second most popular choice being the midwifery staff's inadequacy to provide psychological support. Additionally, with regard to the effect of baseline parameters, we showed that older age, public healthcare postpartum services, and hospitalization during pregnancy were predictors of significantly lower patient satisfaction.

Our results are a confirmation of pre-existing empirical observations, particularly with regard to the disparities between private and public healthcare. Due to the significant effects of the economic crisis and the pandemic, as well as frequent burnout reports amongst Greek healthcare personnel [13,14], midwives serving in public hospitals possess neither the resources nor the personnel to provide the necessary level of individualized care to all women they are attending to. This, combined with the fact that private healthcare has been observed to yield higher patient-reported scores [20] as well as worker-reported scores [21] compared to public healthcare, is indicative of a need for restructuring and rethinking of the approach to midwifery care, in public hospitals in particular, with more emphasis on psychological support and the establishment of a healthier patient-midwife therapeutic relationship. This may be applicable outside of Greece as well, given the international impact of both the economic and pandemic crises on healthcare in general and on midwifery care in particular [22]. This need for restructuring is also supported by the overall lower scores recorded in this study compared to those by Peters et al. (2021) [15], a disparity potentially attributed to differences in resource-setting as well as in the model of care.

Regarding the differences in MMAYpostpartum score between older and younger women, they may be attributed to the increased risk for adverse postpartum outcomes in older patients in general, as well as patient awareness of this risk. Advanced maternal age has been associated with an increased incidence of maternal complications, delivery via caesarean section, preterm delivery, low birthweight neonates, lower average Apgar scores, and several pregnancy conditions and complications, such as ectopic pregnancy, miscarriage, chromosomal abnormalities of the fetus, congenital fetal anomalies, placenta previa, gestational diabetes, and preeclampsia [23]. Furthermore, women in this category are reportedly aware of the increased risks to themselves and their children, with Gossett et al. (2013) [24] demonstrating that 63 to 89% of participants were aware of the associated risks. Awareness of this fact may exert an effect on how the provided midwifery services are perceived during the difficult, transitional postpartum period and thus may be responsible for our results.

Apart from fear and anxiety due to the associated physical risks, several studies have shown that older women are more prone to psychological postpartum disorders, with Aasheim et al. (2012) [25] showing higher scores of psychological distress in older women and Silverman et al. (2017) [26] showing increased postpartum depression rates. Such factors may affect the perceived quality level of midwifery services and thus contribute to the correlation we observed in this study. A similar effect on postpartum psychological status has been observed in patients with pregnancy complications, which frequently require hospitalization, not to mention the effect of hospitalization itself, and thus may explain the significantly lower MMAYpostpartum scores in such patients observed in the present study. Delahaije et al. (2013) [27] observed that pre-eclampsia and other pregnancy disorders, which constitute a frequent reason for hospitalization during pregnancy, had a significant negative impact on psychological health, with anxiety and depression symptoms. Bergink et al. (2015) [28] also showed that pre-eclampsia had a significant effect on postpartum psychiatric pathology incidence (incidence rate ratio: 1.43, 95% CI: 1.22-1.68), while the combination of gestational diabetes and pre-eclampsia had an even more significant effect (IRR 3.86, 95% CI: 1.24-12.00).

The findings of the present study have wider implications for midwifery practice in general and in Greece in particular. They confirm the disparity in quality between public and private healthcare services and indicate the necessity for systematic restructuring of the applied postpartum midwifery care model, with emphasis on continuity of care and psychological support. Additionally, the identified risk factors of older age and a history of hospitalization during pregnancy may be used to identify patients who are in greater need of closer postpartum monitoring and more meticulous midwifery care in lower-resource settings, where such a level of care is not feasible for every woman. Future research on this subject is required, as additional data on the patient perspective would greatly contribute to the improvement of postpartum midwifery care quality. Additionally, a future study may also assess the effect of a more meticulous approach by the midwife, with a focus on psychological support, compared to standard care as a control, with an optional assessment of said effect in the higher-risk patient subgroups identified in the present study, i.e., older women and women with hospitalization histories during pregnancy.

There are a few limitations to the present study that ought to be mentioned. Firstly, the relatively small sample size may have introduced a degree of bias in our findings. Secondly, since no stratification of results based on center of care was conducted, the quality of midwifery services may differ between hospitals and midwives; however, such a thorough assessment was beyond the scope of the present study. Finally, the questionnaire utilized in the present study has not been validated for use on the Greek population; however, since both Greece and Germany, the MMAY validation country, are European Union members and adhere to European healthcare guidelines, the use of the questionnaire was deemed acceptable since no better alternative was available.

## Conclusions

Midwifery care is an indispensable part of overall postpartum care, which has, however, been affected by the recent economic and pandemic crises. Patient perspectives offer a valuable insight into areas in need of improvement as well as into patient subgroups who are in more pressing need of care. In this study, we concluded that postpartum care in public healthcare centers, older maternal age, and history of hospitalization during pregnancy had a statistically significant negative impact on the perceived quality of care and thus may constitute characteristics of patient target groups for more meticulous midwifery care when resource setting prohibits overall improvement of quality. Further research is required to collect additional data on patient insight and to test the present observations in a clinical setting.

## Appendices

The present study was designed and completed in accordance with the Strengthening the Reporting of Observational Studies in Epidemiology (STROBE) Statement [16]. The completed checklist is summarized on Table 4.

**Table 4:** Completed STROBE Checklist.

	Item No	Recommendation	Included	Comment
Title and abstract	1	(a) Indicate the study’s design with a commonly used term in the title or the abstract	Yes	The study is specified to be a cross-sectional study, with the use of a questionnaire in the title: “…using the MMAYpostpartum questionnaire: a cross-sectional study.”
		(b) Provide in the abstract an informative and balanced summary of what was done and what was found	Yes	Details on the setting, participants, the design and a summary of the primary findings are provided within the abstract
Introduction				
Background/rationale	2	Explain the scientific background and rationale for the investigation being reported	Yes	The scientific background and the rational are provided throughout the Introduction section and mainly within the section: “ Midwife contribution is particularly significant… as noted by multiple studies.”
Objectives	3	State-specific objectives, including any prespecified hypotheses	Yes	The specific statement regarding the aims of this paper is provided in “ The aim of this study … compared to large-scale changes) in the Introduction section
Methods				
Study design	4	Present key elements of study design early in the paper	Yes	Key elements of the questionnaire used are provided in the subsection: “Study Design” of the Methods section
Setting	5	Describe the setting, locations, and relevant dates, including periods of recruitment, exposure, follow-up, and data collection	Yes	All relevant information is provided in the subsection: “Setting” of the Methods section
Participants	6	(a) Give the eligibility criteria and the sources and methods of selection of participants	Yes	All relevant information is provided in the subsection: “Participants” of the Methods section
Variables	7	Clearly define all outcomes, exposures, predictors, potential confounders, and effect modifiers. Give diagnostic criteria, if applicable	Yes	All relevant information is provided in the subsection: “Questionnaire” of the Methods section
Data sources/ measurement	8	For each variable of interest, give sources of data and details of methods of assessment (measurement). Describe comparability of assessment methods if there is more than one group	Yes	All relevant information is provided in the subsection: “Questionnaire” of the Methods section
Bias	9	Describe any efforts to address potential sources of bias	Yes	All relevant information is provided in the subsection: “Bias minimization” of the Methods section
Study size	10	Explain how the study size was arrived at	Yes	No pre-specified study size was calculated, as all eligible participants accessible to the investigative team were invited to participate (subsection: “Participants” of the Methods section, lines 5-6)
Quantitative variables	11	Explain how quantitative variables were handled in the analyses. If applicable, describe which groupings were chosen and why	Yes	All relevant information is provided in the subsection: “Questionnaire” of the Methods section
Statistical methods	12	(a) Describe all statistical methods, including those used to control for confounding	Yes	All relevant information is provided in the subsection: “Data collection and analysis” of the Methods section
		(b) Describe any methods used to examine subgroups and interactions	Yes	All relevant information is provided in the subsection: “Data collection and analysis” of the Methods section
		(c) Explain how missing data were addressed	Yes	All relevant information is provided in the subsection: “Data collection and analysis” of the Methods section
		(d) If applicable, describe analytical methods taking account of sampling strategy	N/A	Not applicable
		(e) Describe any sensitivity analyses	N/A	Not applicable
Results				
Participants	13	(a) Report numbers of individuals at each stage of study- eg. numbers potentially eligible, examined for eligibility, confirmed eligible, included in the study, completing follow-up, and analysed	Yes	All relevant information is provided in the “Results” section, first lines
		(b) Give reasons for non-participation at each stage	Yes	All relevant information is provided in the “Findings” section, first lines
		(c) Consider the use of a flow diagram	No	Flow diagram was not included, as there was no control group and tables sufficed as data presentation formats. A flowchart was deemed redundant.
Descriptive data	14	(a) Give characteristics of study participants (eg. demographic, clinical, social) and information on exposures and potential confounders	Yes	All relevant information is provided in the “Results” section, first lines
		(b) Indicate the number of participants with missing data for each variable of interest	Yes	All relevant information is provided in the “Results” section, first lines
Outcome data	15	Report numbers of outcome events or summary measures	Yes	All relevant information is provided in the “Results” section
Main results	16	(a) Give unadjusted estimates and, if applicable, confounder-adjusted estimates and their precision (eg. 95% confidence interval). Make clear which confounders were adjusted for and why they were included	Yes	Table 2, Table 3
		(b) Report category boundaries when continuous variables were categorized	Yes	Table 2
		(c) If relevant, consider translating estimates of relative risk into absolute risk for a meaningful time period	N/A	Not applicable
Other analyses	17	Report other analyses done- eg. analyses of subgroups and interactions, and sensitivity analyses	Yes	All relevant information is provided in the “Results” section, final lines
Discussion				
Key results	18	Summarise key results with reference to study objectives	Yes	All relevant information is provided in the “Discussion” section, first paragraph
Limitations	19	Discuss the limitations of the study, taking into account sources of potential bias or imprecision. Discuss both the direction and magnitude of any potential bias	Yes	All relevant information is provided in the “Discussion” section, last paragraph
Interpretation	20	Give a cautious overall interpretation of results considering objectives, limitations, multiplicity of analyses, results from similar studies, and other relevant evidence	Yes	All relevant information is provided in the “Discussion” section, paragraphs 2,3,4
Generalisability	21	Discuss the generalisability (external validity) of the study results	Yes	All relevant information is provided in the “Discussion” section, the penultimate paragraph
Other information				
Funding	22	Give the source of funding and the role of the funders for the present study and, if applicable, for the original study on which the present article is based	Yes	All relevant information is provided in the manuscript and acknowledgements.
